# MRI brain tumor segmentation using residual Spatial Pyramid Pooling-powered 3D U-Net

**DOI:** 10.3389/fpubh.2023.1091850

**Published:** 2023-02-02

**Authors:** Sanchit Vijay, Thejineaswar Guhan, Kathiravan Srinivasan, P. M. Durai Raj Vincent, Chuan-Yu Chang

**Affiliations:** ^1^School of Electronics Engineering, Vellore Institute of Technology, Vellore, Tamil Nadu, India; ^2^School of Information Technology and Engineering, Vellore Institute of Technology, Vellore, Tamil Nadu, India; ^3^School of Computer Science and Engineering, Vellore Institute of Technology, Vellore, Tamil Nadu, India; ^4^Department of Computer Science and Information Engineering, National Yunlin University of Science and Technology, Yunlin, Taiwan; ^5^Service Systems Technology Center, Industrial Technology Research Institute, Hsinchu, Taiwan

**Keywords:** brain tumor segmentation, 3D U-Net, Spatial Pyramid Pooling, image processing, healthcare

## Abstract

Brain tumor diagnosis has been a lengthy process, and automation of a process such as brain tumor segmentation speeds up the timeline. U-Nets have been a commonly used solution for semantic segmentation, and it uses a downsampling-upsampling approach to segment tumors. U-Nets rely on residual connections to pass information during upsampling; however, an upsampling block only receives information from one downsampling block. This restricts the context and scope of an upsampling block. In this paper, we propose SPP-U-Net where the residual connections are replaced with a combination of Spatial Pyramid Pooling (SPP) and Attention blocks. Here, SPP provides information from various downsampling blocks, which will increase the scope of reconstruction while attention provides the necessary context by incorporating local characteristics with their corresponding global dependencies. Existing literature uses heavy approaches such as the usage of nested and dense skip connections and transformers. These approaches increase the training parameters within the model which therefore increase the training time and complexity of the model. The proposed approach on the other hand attains comparable results to existing literature without changing the number of trainable parameters over larger dimensions such as 160 × 192 × 192. All in all, the proposed model scores an average dice score of 0.883 and a Hausdorff distance of 7.84 on Brats 2021 cross validation.

## 1. Introduction

Brain tumor segmentation using magnetic resonance images (MRI) is a vital step for treating tumors present in the brain and a specialist can use this to find the damage caused by a tumor in a region. The most frequent and severe malignant brain tumors are glioblastomas, often known as gliomas (GBM). Magnetic resonance imaging (MRI) with automated and exact segmentation of these malignancies is critical for early diagnosis as well as for administering and monitoring treatment progression. Assessment of tumor presence is the first step in brain tumor diagnosis and the assessment is done on the basis of segmentation of tumors present in MRI. This process is often done manually making it a time and human intensive task. Moreover, tumors exist in different forms and sizes making it a task requiring expertise. The process of assessment can be sped up by automating the segmentation of brain tumors (1).

The Brain Tumor Segmentation Challenge (BraTS) (2, 3) is a worldwide annual competition that has been concentrating on evaluation of state-of-the-art automated tumor sub-region segmentation algorithms since 2012. The American Society of Neuroradiology (ASNR), the Radiological Society of North America (RSNA), and MICCAI together hosted the BraTS 2021 competition (1) honoring its 10th anniversary. With 1,251 meticulously annotated, multi-institutional, multi-parametric MR images (mpMRI) of patients with various degrees of gliomas, BraTS 2021 provides us with a sizable dataset. The segmentation of the histologically diverse brain tumor sub-regions and the classification of the tumor's O-methylguanine-DNA methyltransferase (MGMT) promoter methylation status are the two main goals of BraTS 2021. In this study, the first task will be the main focus.

**Figure 1:**
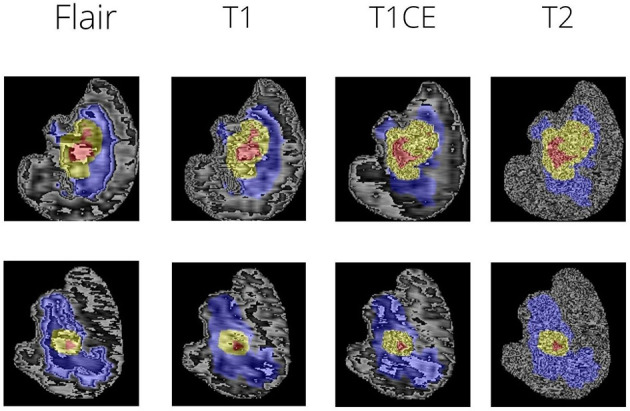
The different views of brain MRI slices with annotations.

The peritumoral edematous/invaded tissue (ED-label 2), the Gd-enhancing tumor (ET-label 4), and the necrotic tumor core are the tumor sub-regions for each patient (NCR-label 1). The peritumoral edematous and infiltrated tissue known as ED has an infiltrative non-enhancing tumor as well as peritumoral vasogenic edema and is linked with an abnormal hyperintense signal envelope on the T2 FLAIR volumes. ET stands for the tumor's enhancing segment and is identified by T1Gd MRI regions that exhibit some enhancement. On T1Gd MRI, the necrotic core of the tumor, or NCR, seems to be substantially less intense. Figure 1 depicts the various tumor sub-regions.

In many vision tasks like segmentation, particularly in the healthcare industry, deep learning-based segmentation systems have shown amazing success, outperforming other traditional methods in brain tumor analysis (4–8). With exceptional results, Fully Convolutional Networks (FCN) (9) achieve end-to-end semantic segmentation for the first time. The most popular architecture for medical picture segmentation is called U-Net (10), which combines a symmetric encoder-decoder topology with skip-connections to maximize information preservation. The performance for image segmentation is greatly improved by many U-Net variants, including U-Net++ (11), two-stage cascaded U-Net (12), and Res-U-Net (13). Although CNN-based techniques have great encoding capacities, because of the convolution kernels' constrained receptive fields, it is challenging to produce an apparent long-distance dependency. Learning global semantic information, which is essential for dense prediction issues like segmentation, is made more difficult by this constraint of convolution operation.

U-Nets consist of residual connections, and these connections are key for reconstruction. These connections pass local and global information to a particular decoder (10). However, information passed from one layer to another may be inadequate for reconstruction. Potentially passing information from a higher resolution may provide better clarity as inputs passed from one layer to another information is lost due to downsizing. Hence skip connections can further be employed to pass information from higher dimensional encoders.

Segmentation maps have been formed using a 3D U-Net which consists of three downsampling and upsampling blocks followed by a set of convolutional layers. The authors use a patching approach to train the model (14). Kaur et al. (15) proposes a 2D and 3D DGA-U-Net. In the 3D model, mainly the pooling layers are replaced with upsampling. The following is done to increase the resolution of the image within the contraction phase of the U-Net. Punn and Agarwal (16) utilized a multi-modal approach to segment brain tumors, where the multi-modalities of the dataset are fused across using deep inception encoding. Finally, a tumor extractor collects features from the fused images to the tumor segmenter. The extractor and segmentation have an U-Net-based architecture. Jiang et al. (12) used a cascaded U-Net in 2 stages. The approach is multi-modal in nature, where in all the class maps are concatenated and passed to the first U-Net. The output of the first U-Net along with the concatenated model input is passed to the second U-Net. Here, a triplet loss is used to train the model, where in the output of the first U-Net along with output of second U-Net and two output maps (Deconvolution and Interpolation approach). Isensee et al. (17) used the nn-U-Net (18) framework to propose a model which is then further enhanced by using post-processing, patching strategies and augmentations that are Brats specific. Qamar et al. (19) increased the contextual information by using a Hyperdense Inception (HI) 3D U-Net. The HI methodology builds the connections between factored convolutional layers to look more like dense connections. U-Nets have been versatile wherein transformer-based models are used within the model (20–22) and have provided significant improvement in results.

Wang et al. (23) proposed a SAR-U-Net which is based on the traditional U-Net with SE (Squeeze and Excitation) block to avoid focus on unnecessary regions within the dataset and Atrous Spatial Pyramid Pooling (ASPP) (24) to pass information on a multi scale basis. The model is trained on LITs dataset and has achieved significant results. Ahmad et al. (25) used a similar approach of using ASPP along with U-Net on Cardiac MRI dataset. The following two approaches are 2-dimensional in nature. Jiang et al. (26) used a 3D Atrous Inception U-Net where the Atrous pooling is used in the residual connections between the encoder and decoder on the Brats dataset. In this approach, the outputs of the succeeding encoder blocks are upsampled and concatenated across before sending to the decoder for reconstruction. Wang et al. (27) introduced the 3D CNN based Transformers for segmenting brain tumors.

Hence, we were able to identify some research gaps:

- As can be seen, existing literature uses heavy approaches such as the usage of nested and dense skip connections and transformers. Hence an approach which considers the parameters in mind is needed. Considering applications such as edge computing which heavily emphasize efficient and accurate predictions, the proposed mechanism fits such problem statement in hand.
- Moreover, the skip connections have always been an aspect of the experimentation. Additional information to the decoder layers through mechanisms such as ASPP has given performance improvement. Hence utilizing a similar mechanism on multiple encodings in a 3-dimensional manner seemed to be an idea for the research.

### 1.1. Contributions

We propose a U-Net with SPP and attention. SPP takes information from three encoder layers and passes it to the decoder in the U-Net. The proposed addition provides the model with additional context and information for better reconstruction by providing scope from neighboring layers. The proposed mechanism does not have additional training parameters therefore the need for computational power remains the same. Therefore, the resultant model is lightweight in nature aiding for faster medical diagnosis and medical workflow in a production environment. To introduce reproducibility, the codebase utilized has been made public: https://github.com/sanchitvj/rsppUnet-BraTS-2021. We encourage the community to use and possibly improve the mechanism further in the form of open-source contributions.

## 2. Materials and methods

### 2.1. Data processing

The dataset used was Brats 2021. The MRI scans were firstly bought across to a common dimension of 160 × 192 × 192. This size was arrived upon based on experimentation and the comparison was done on the basis of Dice Score (further discussed in results). Figure 1 shows sample MRI slices from two MRI files. The scans are brought to a common dimension using padding and cropping. Padding is used whenever the image size is lower than the specified size and in cases where dimension of the original image being larger cropping takes place. Augmentations are key in this case as the number of data samples is low, hence a combination of augmentations are used at random. The following augmentations are used:

- Image flip
- Brightness adjust
- Rotation: Images can be rotated on the *z*-axis with the maximum angle of rotation being 30° and the minimum angle of rotation being −30°.
- Elastic transformation
- Intensity shift

Note that the choice of augmentations, within this set, used are random hence this makes the model robust to overfitting. The following is achieved by randomly choosing the augmentations on the basis of a threshold. *K*-Folds were used to divide the data into 5-folds, with Fold 1 being used to assess the model's performance and the other folds being used for training. Table 1 demonstrates the distribution of the data used. Fold 1 was chosen on the basis of metric stability. It was often noted that results achieved on Fold 1 had a relatively smooth progression. This dataset has a balance of noisy and normal data samples. In a way, training on these other noisy folds makes the model get a generalized understanding of the data.

**Table 1:** Data split for brats 2021.

**Data split**	
**Split name**	**Number of samples**
Train split	1,000
Validation split	250

### 2.2. Residual spatial pyramid pooling-powered 3D U-Net model

Spatial Pyramid Pooling (21) has been widely used in classification and object detection. The reason being, SPP provides an effective representation of varying sized images and it can be considered as an ensemble of pooling layers. In this way, the feature maps captured by convolutional layers can be deciphered in various ways, and pooling has often been the solution to aggregate the learning of convolutional layers. Hence, concentrating information using different dimensional pooling layers can provide representations that can further enhance the performance of the model.

Atrous Spatial Pyramid Pooling was proposed based on SPP and carries the concept of SPP by using parallel Atrous Convolutional layers. ASPP has been extensively used in semantic segmentation, and it serves the purpose of providing context at different levels or views. ASPP has been employed in various studies within brain segmentation. However, as per Tampu et al. (28), boosting context alone does not increase the performance of the model.

Attention is a process through which we humans put forth focus on doing certain tasks. While reading, we capture context by understanding neighboring words within a sentence. This mechanism is applied to the attention layer and its purpose is to capture context. The attention layer has been extensively employed in deep learning and has contributed to cutting-edge outcomes. Attention is obtained for the model by combining the output of two encoder layers. By feeding the output of two encoder layers into two different 3D convolutional layers, the following is accomplished. The output of the two layers is combined, and relu is then used to activate it. The activated output is passed through a 3D convolutional layer and is then normalized and activated. Fusing the output of these layers along with activation aids in maintaining context while not compromising on the dimensionality aspect.

**Figure 2:**
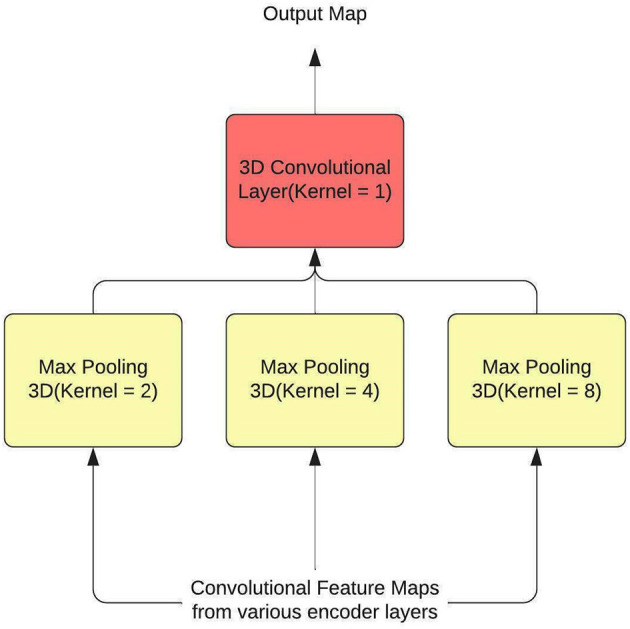
The architectural diagram for SPP.

Hence, SPP is used as a feature aggregator within the model, and to introduce context, attention layers are employed. The SPP layer, along with the attention layer, have been used to replace some residual connections within the U-Net. SPP is typically used at the end of the process, after the feature maps have been flattened so that fully connected neural networks can use the maps to predict class(es) or bbox(es). A 3D convolutional layer with a kernel size of 1 is utilized to modify SPP so that it functions as a residual connection. The output of the SPP is again converted to a 3D representation by this layer. Additionally, by sending input from many encoder levels to each pooling layer, information is gathered over a wide range. Figure 2 shows the architecture of the SPP Layer.

The U-Net used is based on the NvNet (29) and the following figure shows the architecture of the model. As shown in the architecture SPP is just used in two places, the reason for the same was to maintain the aspect of dimensionality. The SPP layer takes input from various encoder layers therefore when pooling is applied the dimension of the output varies substantially.

**Figure 3:**
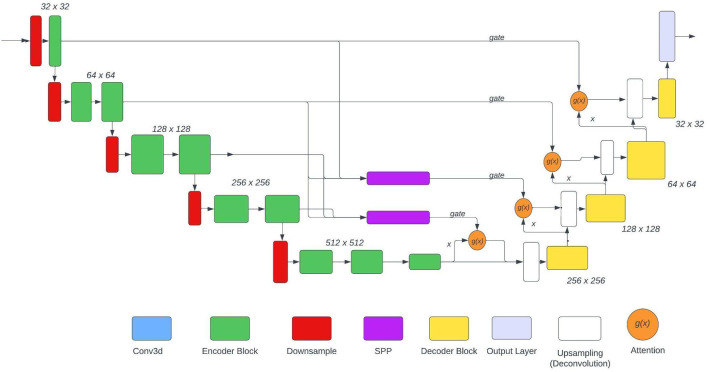
The architecture of U-Net used.

In this paper, experimentation is done on 3 model architectures based on Figure 3:

- No SPP: The SPP blocks would be omitted therefore boosting the model only in terms of context.
- 1 SPP: The upper SPP block would be removed from the architecture keeping only the lower block (with 3rd encoder layer). Hence boosting the model with a combination of context and features.
- 2 SPP: Both the SPP blocks were used. This model carries more feature boost from the other two models used.

#### 2.2.1. Training procedure

Table 2 shows the hyperparameters used during training. In general, the increase in performance post 60 epochs was negligible hence the same was chosen. We experimented with different sizes (image size format: channel ^*^ length ^*^ width) such as 160 × 160 × 160, 128 × 160 × 160, and 160 × 192 × 192. The original dimensions of the slice were 155 × 240 × 240. Among these 160 × 192 × 192 showed the best convergence so we decided to go with it. The optimizer of choice was kept as Adam and the loss function of choice was Squared Soft Dice Loss as proposed by Milletari et al. (30) the working of the same shown in Equation (1). Here *p*_*i*_ denotes the truth label for the pixel and *g*_*i*_ denotes the model prediction where *N* denotes the number of voxels. The prime reason for choosing this function was to avoid the focus of the loss function from the background regions. In problems such as brain segmentation, the size of the regions consisting of tumors are very small relative to the background region and weighted losses have not been the most efficient solution for the same. This function ranges in the value of 0–1 with an objective to maximize the loss.


(1) $$\begin{array}{l} Dice\;Loss=\frac{2^{*}\overset{N}{\underset{i}{\sum }}p_{i}g_{i}}{p_{i}^{2}g_{i}^{2}} \end{array}$$

Based on our experimentation we found the issue of gradient explosion hence group normalization was employed. Batch Normalization did not work in our cases as high batch size could not be used for training. Batch size >1 did not provide the expected results and at times would also result in the GPU running out of memory and process killing. Hence, the choice for batch size was kept as 1. A Nvidia Tesla V100 GPU was utilized for the training of the models.

**Table 2:** Hyperparameters used for training.

**Hyperparameters used**	
**Hyperparameter**	**Value**
Image size (channels ^*^ length ^*^ width)	160 × 192 × 192
Epochs	60
Learning rate	2.50E-04
Weight decay	1.00E-07
Scheduler	Cosine annealing LR
Criterion	Dice loss
Optimizer	Adam
Normalization	Group norm

#### 2.2.2. Training procedure

The evaluation process of the model has been done on the basis of cross-validation and the model was evaluated on two metrics:

- Dice Score (as showing in Equation 2): In short it is the F1-Score conveyed on behalf of image pixels: Wherein the ground truth is the annotated pixels. Dice score is an efficient metric as it penalizes false positives: If the predicted map has large false positives, it is used in the denominator rather than the numerator.

(2) $$\begin{array}{l} Dice\;Score=\frac{2{\;}^{*}Region\;of\;Overlap}{Region\;of\;Union} \end{array}$$

- Hausdorff Distance: The Hausdorff distance (31) describes how closely each point in a model set resembles a point in an image set and vice versa. So, the degree of similarity between two items that are superimposed on one another can be gauged using this closeness.

It should be noted that Hausdorff Distance is unconcerned with the size of the image's background. By calculating the extensive distance between the extremes of the two outlines, the Hausdorff distance complements the Dice metric. A prediction may show nearly voxel-perfect overlap since it severely penalizes outliers, but the Hausdorff distance will only be meaningful if a certain voxel is far from the reference segmentation. This statistic is quite useful for determining the clinical importance of segmentation, despite being noisier than the Dice index.

## 3. Results and discussion

Two models from the Brats 2021 dataset, as well as one model from each of the Brats 2020 and Brats 2019 datasets, are compared to the suggested model.

As per Table 3 the following inferences can be made:

- The model with 1 SPP block performed the best amongst the architectures proposed. Therefore, it can be deduced that the boosting of context and features go hand in hand.
- In the case of Hausdorff Distance, all the three models have the lowest metric when the class is Enhancing Tumor.
- In the case of Dice Score, both No SPP and 2 SPP models achieve similar results in Whole Tumor and Tumor Core. Both the models outperform 1 SPP in Whole Tumor however lose out to 1 SPP in Tumor Core. All the models achieve the lowest Dice Score in the case of Enhancing Tumor.
- When comparing models trained on Brats 2019 and Brats 2020, the proposed work outperforms the model however this is a general trend.
- With respect to Brats 2021, the proposed work gives comparable results to Hatamizadeh et al. (32) however loses out to Jia and Shu (22) on a large margin. One thing to note, both of these models use transformers which naturally provide more context and features. Transformers are heavy on parameters, while the proposed approach requires no extra parameters.

**Table 3:** Results obtained in standard setting.

**Model name**	**Metrics**							
	**WT, whole tumor; TC, tumor core; ET, enhancing tumor (sub regions of tumor affected brain)**							
	**Hausdorff distance**				**Dice**			
	**WT**	**TC**	**ET**	**Average**	**WT**	**TC**	**ET**	**Average**
No SPP	13.070	11.010	10.210	11.430	0.908	0.877	0.838	0.870
1 SPP	9.430	7.780	6.300	7.840	0.899	0.899	0.850	0.883
2 SPP	16.060	5.650	5.270	8.990	0.904	0.880	0.845	0.876
Hatamizadeh et al. (32)	4.739	15.309	16.326	12.120	0.927	0.876	0.853	0.890
Jia and Shu (22)	3.000	2.236	1.414	2.220	0.926	0.935	0.887	0.920
Qamar et al. (19)	–	–	–	–	0.875	0.837	0.795	0.840
Jiang et al. (12)	4.610	4.130	2.650	3.800	0.888	0.837	0.833	0.850

The model was also trained on image size of 160 × 160 × 160. This was done to understand the impact of a smaller image size. Table 4 conveys the same.

**Table 4:** Results obtained with image size 160 × 160 × 160.

**Model name**	**Metrics**							
	**WT, whole tumor; TC, tumor core; ET, enhancing tumor (sub regions of tumor affected brain)**							
	**Hausdorff distance**				**Dice**			
	**WT**	**TC**	**ET**	**Average**	**WT**	**TC**	**ET**	**Average**
No SPP	34.1	7.97	7.13	16.4	0.895	0.872	0.837	0.868
1 SPP	18.6	6.13	4.88	9.87	0.887	0.879	0.842	0.869
2 SPP	20.12	7.42	6.22	11.25	0.886	0.876	0.843	0.868

The following inferences can be made from Table 4:

- 0.01 was the difference in average dice score between the models trained on different image sizes. However, a significant difference was observed in the Hausdorff distances.

- Again 1 SPP model performed the best but the margin of difference was next to none. Hence we can infer that a high image size is a key contributor to increase performance when SPP is utilized and the following inference proves the point of passing higher resolution features through residual connections.

**Figure 4:**
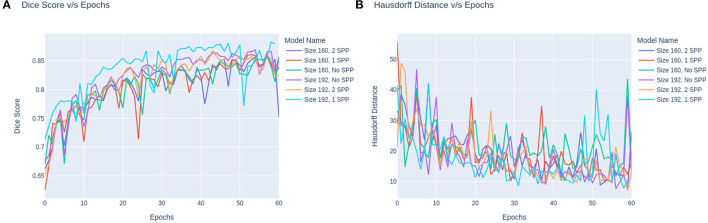
**(A)** The dice score vs. epochs. **(B)** Hausdorff scores vs. epochs.

The trend in the metrics can be seen as shown in Figure 4. Figure 4A represents the Dice Score vs. Epochs and Figure 4B portrays the Hausdorff Scores vs. Epochs. Regardless of the training image size, the models carry a similar trend where the dice scores plateau at 60 epochs. Secondly, the performance of models trained on 160 × 160 × 160 are lower than the models trained on 160 × 192 × 192. Moreover, it can also be observed that the convergence of the loss is delayed for models with SPP. Although SPP does not bring any extra trainable parameters it still keeps the model from converging. Based on the trend, the model can be fine-tuned at extremely small magnitudes of learning rates to increase the performance of the model. In the case of Figure 4B it can be observed that models without SPP tend to provides metric stability once the model reaches the last few epochs.

**Figure 5:**
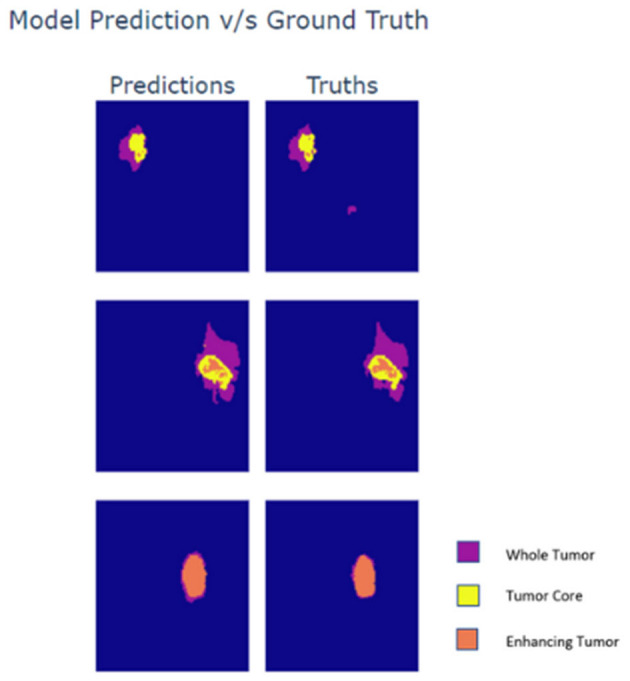
Prediction vs. ground truth segmentation mask comparison.

Lastly, the output maps from the model are analyzed in Figure 5. For the analysis three slices from different MRI scans are taken wherein each slice exhibits varying presence of class. The first row within the plot contains a sparse volume of enhancing tumor, while the second row contains a moderate volume of enhancing tumor. The last row majorly contains enhancing tumors. Based on the predictions it can be observed that the model is able to predict all types of cases with great accuracy. The reason for the visualization is to showcase the model's ability to predict enhancing tumors accurately as its presence in data is limited. Moreover, the model is also able to detect abnormal whole tumor shapes with ease which conveys that the model is fit for real world diagnosis. In the first scan the model is able to predict sparse presence of enhancing tumors which is very crucial.

## 4. Conclusion

We propose U-Net with SPP and Attention Residual Connections in this work. The proposed model attachment is a lightweight mechanism which boosts information and context in the model by passing high and low resolution information to the decoders in the Unet. The proposed mechanism is applied to the NvNet model in varying frequencies which then produces different variants: Model with attention, Model with attention and 1 SPP, and Model with attention and 2 SPP. The model with 1 SPP and attention performs the best and provides comparable results to heavy models with transformer residual attachments. The average Dice Score and Haussdorf distance for the model with 1 SPP and attention are 0.883 and 7.99, respectively. The proposed mechanism is an approach to boost information and context hence giving considerable performance boosts. This approach plays well in applications such as edge computing which requires a balance of computational efficiency and performance. Such an approach could be utilized in mobile healthcare stations which need immediate diagnosis with less computation power. However, the impact of performance improvement at times falls a bit short compared to heavy approaches and it boils down to the extra trainable parameters brought by the components which eventually capture more patterns. In the current work, the mechanism is only adapted to one particular model and in the future, we aim to make the mechanism adaptable to various other 3D-Unet architectures.

## Data availability statement

The original contributions presented in the study are included in the article/supplementary material, further inquiries can be directed to the corresponding author.

## Author contributions

KS and C-YC conceptualized and supervised the research, carried out the project administration, and validated the results. SV and TG contributed to the development of the model, data processing, training procedures, and the implementation of the model. SV, TG, and KS wrote the manuscript. SV, TG, KS, PV, and C-YC reviewed and edited the manuscript. C-YC carried out the funding acquisition. All authors contributed to the article and approved the submitted version.
